# The relationship between carbonic anhydrase IX (CAIX) and patient survival in breast cancer: systematic review and meta-analysis

**DOI:** 10.1186/s13000-023-01325-9

**Published:** 2023-04-15

**Authors:** Suad A. K. Shamis, Joanne Edwards, Donald C. McMillan

**Affiliations:** 1 Academic Unit of Surgery, School of Medicine, University of Glasgow, Royal Infirmary, Alexandria Parade, Glasgow, G31 2ER UK; 2 Unit of Molecular Pathology, School of Cancer Sciences, University of Glasgow, Wolfson Wohl Cancer Research Centre, Garscube Estate, Switchback Road, Glasgow, G61 1QH UK

## Abstract

**Purpose:**

Hypoxia is a characteristic of many solid tumours and an adverse prognostic factor for cancer therapy. Hypoxia results in upregulation of carbonic anhydrase IX (CAIX) expression, a pH-regulating enzyme. Many human tissue studies have examined the prognostic value of CAIX expression in breast cancer but have yielded inconsistent results. Therefore, a systematic review and meta-analysis was undertaken to assess the prognostic value of CAIX expression for breast cancer patients.

**Methods:**

The electronic databases were systematically searched to identify relevant papers. The clinical outcomes included disease-free survival (DFS), recurrence-free survival (RFS) and overall survival (OS) in breast cancer patients. Review Manager version 5.4 was employed to analysis data from 23 eligible studies (containing 8390 patients).

**Results:**

High CAIX expression was associated with poorer RFS [HR = 1.42, 95% CI (1.32−1.51), *p* < 0.00001], DFS [HR = 1.64, 95% CI (1.34−2.00), *p* < 0.00001], and OS [HR = 1.48, 95% CI (1.22−1.80), *p* < 0.0001]. Heterogeneity was observed across the studies. There was an effect of the CAIX antibody employed, scoring methods, and tumour localisation on CAIX expression.

**Conclusion:**

CAIX overexpression was significantly associated with poorer RFS, DFS, and OS in breast cancer patients. However, further work in high quantity tissue cohorts is required to define the optimal methodological approach.

## Introduction

Breast cancer is one of the most common cancers prevalent across the world, and is one of the leading causes of morbidity and mortality in women [1]. Hypoxia is a prominent feature of the tumour microenvironment in a variety of common solid tumours as a result of an imbalance between the increasing demand for oxygen and nutrients by proliferating cancer cells and an inadequate blood supply resulting from impaired angiogenesis in the tumour microenvironment [2]. Hypoxic conditions may result in focal expression of hypoxia inducible factor 1α (HIF-1α), a key regulator of the hypoxia response [3]. Hypoxia-associated enzyme carbonic anhydrase IX (CAIX) is a direct transcriptional target of HIF-1α and is one of the most commonly upregulated genes in response to hypoxia. Since HIF-1α expression is transient and CAIX expression less transient, CAIX expression is a robust biomarker of tumour hypoxia [4, 5].

CAIX, is one of 15 carbonic anhydrase (CA) isoforms reported in humans and has been described as a homodimeric transmembrane glycoprotein. The domain structure of mature CAIX contains a proteoglycan-like domain, a catalytic domain, a transmembrane domain, and a cytoplasmic tail [6]. CAIX facilitates the reversible hydration of carbon dioxide to bicarbonate and protons [4]. Thus, it plays a major role in maintaining the pH gradient between cells and their extracellular space [7]. CAIX is normally expressed in few tissues including the gut epithelium and biliary tree [8, 9] but appears to be upregulated in response to tumour hypoxia in many tumour types including breast cancer [10, 11].

The majority of studies in the literature suggest that CAIX can serve as a biomarker and therapeutic target in different tumour types [12]. Published breast cancer data supports CAIX as a marker of aggressive tumour behaviour, and high CAIX expression correlates with high tumours grade [13–16] and loss of ER and PR expression [10, 13–15, 17]. CAIX has also been reported to be positively associated with necrosis [18], larger tumour size and basal-like tumours [15, 19]. High expression of CAIX is independent prognostic factor in ER-positive breast cancer [20]. Furthermore, overexpression of CAIX protein in TNBC is associated with a BRCA1 mutant signature and loss of BRCA1 function [21]. Several studies have reported that CAIX overexpression in breast cancer is a poor prognostic marker for distant metastasis and survival [13, 15, 19, 22, 23], however, in contrast, several other studies did not report a significant association with RFS or OS [14, 24–26]. Studies have reported that CAIX expression was associated with worse prognosis for TNBC patients [15, 21], however, Ozretic et al. [25] reports no association with TNBC survival. It seems likely that these contradictory findings at least partially may be explained by its differential expression in various subtypes of breast cancer, power of the studies and techniques employed to assess expression levels [15, 19].

It is of interest that a meta-analysis of CAIX in renal cell carcinoma showed that high CAIX expression was associated with an improved OS [27]. In contrast, a meta-analysis in head and neck cancer patients concluded high CAIX expression was associated with poorer OS and DFS [28]. A meta-analysis of the association between CAIX expression and outcome in breast cancer has not been performed. The aim of this meta-analysis of published clinical studies is therefore to elucidate the prognostic value of CAIX expression in breast cancer patients.

## Materials and methods

### Search strategy

The present review was performed according to guidelines for systematic reviews and meta-analysis of tumour marker prognostic studies. To identify all potentially relevant studies, the author (SS) searched electronic databases (Google Scholar, PubMed and Web of Science) to obtain all relevant articles about CAIX as a prognostic factor for breast cancer patient survival using the following search terms: “breast cancer” or “breast carcinomas” or breast neoplasm”, “CAIX” or “carbonic anhydrase-IX” “prognosis” or “survival” or “outcome”, without language limitations. The bibliographies of the included studies were also searched to identify additional studies.

### Study selection

Studies were considered eligible if they fulfilled the following criteria: (1) were in breast cancer; (2) determined CAIX expression in breast cancer using immunohistochemistry (IHC); (3) examined the relationship between CAIX expression and clinical outcome; (4) provided sufficient data to estimate hazard ratios for survival rates and their 95% confidence intervals. The studies were excluded if they were: (1) not in English; (2) animal studies; (3) cell culture-based studies; (4) had insufficient data for analysis or critical information that could not be extracted.

### Data extraction

Three investigators (SS, DM and JE) screened eligible studies and extracted the following information: name of first author, year of publication, country, sample size, detection method, expression pattern, scoring method, threshold values, cellular localization, and clinical endpoints. Furthermore, hazard ratio and their corresponding minimum and maximum 95% CIs were also collected for RFS, DFS, and OS if reported in the text. If both univariate analysis and multivariate analysis were used in a given study, the survival data of multivariate analysis were preferably included. Survival curves were used to extract data to estimate HR when it was not possible to extract HR directly from the article following the method of Tierney et al. [29].

From this search, the titles and abstracts of articles were initially examined to determine the relevance of these publications. Then, the full texts of the remaining articles were obtained and carefully reviewed. The reference lists of all relevant articles were also examined manually to identify additional studies that may not have been identified by the strategy outlined above. Discrepancies between the reviewers were resolved by discussion.

### Statistical analysis

RFS was the length of time from either the date of diagnosis or the start of therapy to the date of the first loco‐regional or systemic recurrence. DFS was evaluated as the time from the date of the initial curative surgery to the date of the first loco‐regional or systemic relapse, or mortality in the absence of relapse. OS was defined from the day of surgery until death of the patient either from cancer or a cause other than breast cancer.

The meta-analysis was performed using Review Manager (RevMan) version 5.4 (The Nordic Cochrane Centre, The Cochrane Collaboration, Copenhagen, Denmark). The pooled effects were estimated using HRs and 95% CIs for prognostic data to evaluate the associations between CAIX and breast cancer survival. Heterogeneity among the studies was assessed by using the Cochran Q test and Higgins I^2^ statistics. A significant heterogeneity was considered at I^2^ > 50% and subsequently a random effect model should be applied. If not, a fixed effect model was used. Significant relationships were estimated at a *p* value < 0.05.

## Results

### Studies selection process

The search yielded 1294 articles in Google scholar, 1079 articles in PubMed and 84 articles in Web of Science. After removal of 530 duplicates, 1927 unique articles were left for evaluation. Of these, 1620 articles were excluded based on title and abstract, and 307 remaining articles were identified through full paper review. Subsequently, 284 studies were excluded for the following reasons: 181 lacked survival outcomes, 60 were animal studies, and 35 were cell line studies, 5 were non IHC based methods, two of them were review or meta-analysis, and one was non-English studies.

**Fig. 1:**
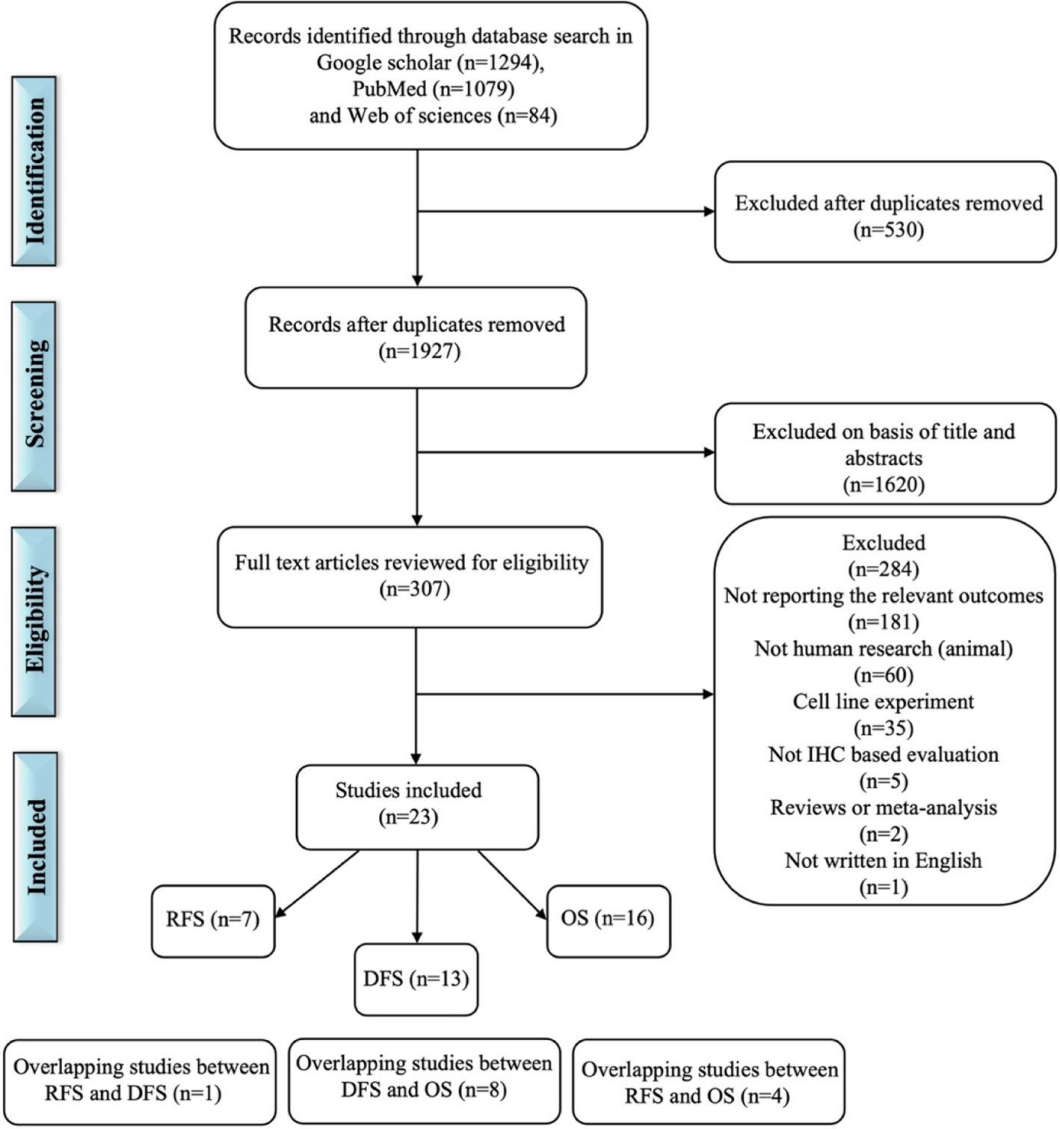
Flow chart of selecting articles describing the association between CAIX expression and patient’s prognosis

The reference list of each study was examined and did not identify any further studies for inclusion in this analysis. Finally, a total of 23 independent studies from 15 different countries were considered eligible for inclusion in the meta-analysis. The study flow diagram is shown in Fig. 1.

### Study characteristics

A total of 23 studies involving 8390 participants addressing CAIX expression in breast cancer met the criteria for this review and the characteristics of eligible studies are summarised in Tables 1, 2, 3 and 4. The majority of studies were carried out in early stage breast cancer and mainly in patients with ductal disease with minimum and maximum sample sizes of 40 and 3630 respectively. Most of the studies reported the length of the follow-up period, and 13 of them exhibited a sufficiently long follow-up (defined as a median follow-up time > 60 months) for the outcomes to be determined.

IHC methodology varied between the studies. Four different antibodies were used. Also, different localizations for protein expression and different quantification methods were reported. Thresholds have been applied to stratify patients into groups with low and high tumour CAIX expression and varied among the studies from 1 − 10% or a score of 1 − 52.5.

### Quantitative data synthesis

**Fig. 2:**
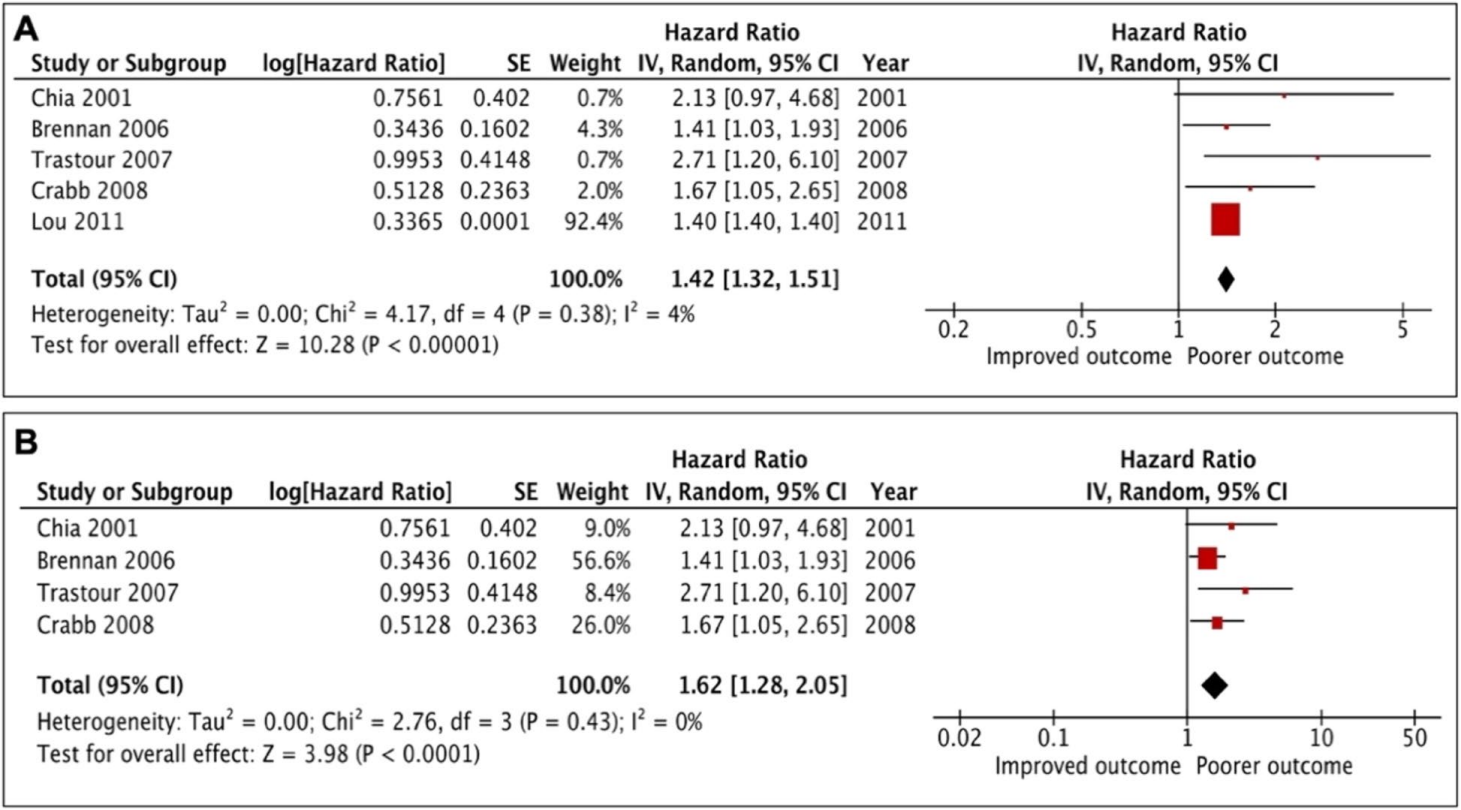
Forest plot for the relationship between CAIX expression and recurrence free survival in breast cancer patients. Including Lou’s study [**A**], after excluding Lou’s study [**B**]

**Fig. 3:**
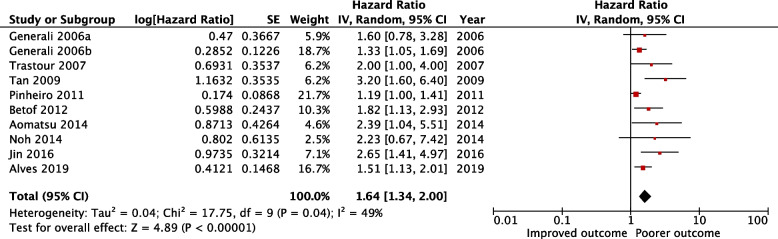
Forest plot for the relationship between CAIX expression and disease-free survival in breast cancer patients

**Fig. 4:**
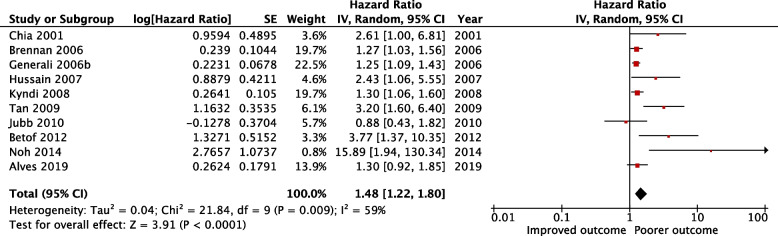
Forest plot for the relationship between CAIX expression and overall survival in breast cancer patients

The pooled HR and 95% CI was calculated according to survival data including RFS, DFS, and OS. Studies with small number of patients < 100 were excluded from the analysis (*n* = 3). The detailed results were provided in Tables 1, 2 and 3 and the forest plots were provided in Figs. 2, 3 and 4.

### Analysis of CAIX expression and RFS

Recurrence free survival was reported in 7 studies, of which one study provided incomplete data to estimate the HR and was therefore not included in the analysis (Table 1). One study was also excluded from the analysis because of small sample sizes. In the remaining 5 studies (n = 4578), patients with high tumour CAIX expression had a significantly worse RFS [HR = 1.42, 95% CI (1.32 − 1.51), *p* < 0.00001], with mild non significant heterogeneity (I^2^ = 4%, *p* = 0.38) (Fig. 2a and 2b). 3630 participants from 4578 was came from the report of Lou and co-workers [19]. Thus, further analysis was performed with this study excluded and the result was proven to be stable, the exclusion of this report did not significantly alter the results [HR = 1.62, 95% CI (1.28 − 2.05), *p* < 0.0001] and no heterogeneity was shown (I^2^ = 0%, *p* = 0.43) (Fig. 2b).

**Table 1:** Studies characteristics and the impact of CAIX on recurrence free survival

Author(s)	Country	Patients (n)	Median follow up (months)	Cancer death n (%)	Subtype	Antibody for IHC/ dilution	Scoring method	High CAIX expression n (%)	Score range and location	Definition of positive	Tumour stage	Multivariate variables	Hazard ratio (95% CI)	*p-*value	HR estimation
**Beketic-Oreskovic** **et al. 2011 **[23]	Croatia	40	55.8	24 (60)	Mixed BC	NA 1:100	Percentage and intensity	24 (60)	Score1-3 Membranous and cytoplasmic	Score > 52.5	NA	Necrosis, tumour size, LN, histological grade and CAIX expression	MV analysis 3.99 (1.38–11.59)	0.011	Reported in text
**Lou et al. 2011 **[19]	Canada	3,630	126	NA	Mixed BC	M75 1:50	Absent or present	566 (16)	0–1 Location not stated	Score ≥ 1	NA	NA	UV analysis 1.4	< 10` ^17^	Reported in text
**Lancashire et al. 2010 **[48]	United Kingdom	244	67	NA	Mixed BC (*n* = 160)	Abcam 15,086 1:2,500	Absent or present	29 (18)	0–1 Membranous	Score ≥ 1	NA	NA	UV analysis NA	0.097	Reported in text
**Crabb et al. 2008 **[49]	Canada	313	NA	NA	Mixed BC	M75 1:50	Absent or present	47 (15)	0–1 Location not stated	Score ≥ 1	NA	ER, PR, Her-2 status, EGFR, Ki67, p53, CK5/6 and CAIX expression	MV analysis 1.67 (1.06–2.64)	0.03	Reported in text
**Trastour et al. 2007 **[16]	France	132	138	20 (15)	Mixed BC	MN75 1:10,000	Percentage	38 (29)	0–100 Membranous	> 1%	NA	Tumour size, LN status, tumour grade, HIF-1a and CAIX expression	MV analysis 2.7 (1.2–6.1)	0.01	Reported in text
**Brennan et al. 2006 **[50]	Sweden	400	166.8	NA	Mixed BC LN + ve (n = 104) premenopausal	M75 1:2000	Absent or present	42 (11)	0–1 Membranous	Score ≥ 1	II	NA	UV analysis NA	0.032	Reported in text
**Chia et al. 2001 **[13]	United Kingdom	103	74.4	32 (31)	Mixed BC	M75 1:50	Percentage and intensity	49 (48)	Score 0–300 Membranous	Score ≥ 1	NA	LN status, grade, size, ER, necrosis and CAIX expression	MV analysis 2.13	0.06	Reported in text

Table detailing papers which investigated the prognostic role of CAIX on RFS

*IHC* Immunohistochemistry, *BC* Breast cancer, *LN* Lymph node, *ER* Oestrogen receptor, *PR* Progesterone receptor, *Her-2* Human epidermal growth factor receptor-2, *EGFR* Epidermal growth factor receptor, *CAIX* Carbonic anhydrase IX, *UV* Univariate analysis, *MV* Multivariate analysis, *HR* Hazard ratio, *95%CI *95%confidence interval, *NA* Not available

Since few studies examined the association between tumour CAIX expression and RFS (n = 5), subgroup analysis was not carried out. The majority of studies were associated with poor prognosis and similar antibodies were used.

### Analysis of CAIX expression and DFS

Effect of CAIX expression on DFS in breast cancer could be evaluated in 13 studies (n = 2356 patients). Due to a small observational number, one further study was excluded from the analysis. The complete data to estimate the HR could not be retrieved from two studies and were therefore not included in the analysis. HR for 3 studies was calculated from available numerical data (Table 2). Overall, high CAIX expression in 10 studies (n = 1882) was associated with a worse DFS, [HR = 1.64, 95% CI (1.34–2.00), *p* < 0.00001]. Mild heterogeneity was detected across these studies (I^2^ = 49%, *p* = 0.04) (Fig. 3). Therefore, subgroup analysis was performed to explore the potential sources of heterogeneity based on survival analysis, study region, antibodies used, cellular localization, and scoring methods.

**Table 2:** Studies characteristics and the impact of CAIX on disease-free survival

Author(s)	Country	Patients (n)	Median follow up (months)	Cancer death n (%)	Subtype	Antibody for IHC/ dilution	Scoring method	High CAIX Expression n (%)	Score range and location	Definition of positive	Tumour stage	Multivariate variables	Hazard ratio (95% CI)	*p-*value	HR estimation
**Alves et al. 2019 **[26]	Brazil	196	73.9	NA	Mixed BC	ab15086 1:200	Percentage and intensity	13 (7)	Score 0–6 Membranous	Score > 2	T1-4 N0-3 M0	NA	UV analysis NA	0.005	Reported in text
**Jin et al. 2016 **[45]	South Korea	270	NA	NA	TNBC	Abcam 1:75	Percentage	59 (22)	1–100 Membranous and cytoplasmic	≥ 10%	I-II	LVI, LN metastasis, HIF-1α, CAIX and combined HIF-1α and CAIX expression	MV analysis 2.65 (1.41–4.97)	0.002	Reported in text
**Aomatsu et al. 2014 **[51]	Japan	102	6.2	NA	Mixed BC	M75 1:1000	Intensity	47 (46)	Score 0–3 Membranous	Score ≥ 2	IIA-IIB or IIIA	ER, PR, molecular subtypes (HR + /Her-2) and CAIX expression	MV analysis 2.39 (1.04–5.49)	0.041	Reported in text
**Noh, Kim, and Koo 2014 **[44]	South Korea	334	NA	31 (9.3)	AR + /ER − BC	Abcam 1:100	Percentage and intensity	96 (29)	Score 0–6 Location not stated	Score ≥ 2	T1-3	T stage, LN status, histologic grade and CAIX expression	MV analysis 2.23 (0.67–7.43)	0.191	Reported in text
**Choi, Jung, and Koo 2013**	South Korea	276	67	23 (8)	Mixed BC	NA 1:100	Intensity	90 (33)	Score 0–3 Cytoplasmic	Score ≥ 2	T1-3 N0-3	-	UV analysis NA	0.271	Reported in text
**Currie et al. 2013 **[52]	New Zealand	87	NA	NA	Mixed BC	Novus Biologicals 1:1000	Percentage and intensity	43 (49)	Score 0–8 Membranous	Score ≥ 3	NA	-	UV analysis NA	0.47	Reported in text
**Betof et al. 2012 **[53]	USA	209	99.6	32 (16.6)	Mixed BC based on CT	M75 NA	Percentage and intensity	182 (88)	Score 0–300 Membranous and cytoplasmic	Score ≥ 50	NA	NA	UV analysis 1.82	0.014	Reported in text
**Kaya et al. 2012 **[54]	Turkey	111	110	NA	Group1:HR + , Her-2-ve Group2:HR-, Her-2 + ve	H-120 1:100	Absent or present	62 (65)	0–1 Membranous	Score ≥ 1	T1-4 N0-3 M0	-	UV analysis NA	0.344	Survival curve
**Pinheiro et al. 2011 **[17]	Portugal and Brazil	122	NA	NA	Mixed BC	ab15086 1:2000	Percentage and intensity	22 (18)	Score 0–6 Membranous	Score ≥ 3	T1-3	NA	UV analysis NA	0.045	Survival curve
**Tan et al. 2009 **[15]	UK and Australia	182	131.9	99 (21.7)	Mixed BC Patients treated with CT (n = 182)	NA	Percentage	59 (14)	0–100 Membranous	≥ 10%	NA	NA	UV analysis 3.20 (1.79–5.70)	< 0.001	Reported in text
**Trastour et al. 2007 **[16]	France	132	138	20 (15)	Mixed BC	MN75 1:10,000	Percentage	38 (29)	0–100 Membranous	> 1%	NA	Tumour size, LN status, tumour grade, HIF-1α and CAIX expression	MV analysis 2 (1.0–4.2)	0.05	Reported in text
**Generali et al. 2006a **[55]	Italy	166	53	22 (11.7)	Mixed BC	M75 1:50	Intensity	41 (24.7)	Score 0–2 Location not stated	Score ≥ 1	T2-4 N0-1	NA	UV analysis NA	0.02	Reported in text
**Generali et al. 2006b **[10]	Italy	169	NA	21 (12.5)	Mixed BC	M75 1:50	Intensity	41 (24)	Score 0–2 Location not stated	Score ≥ 1	T2–4 N0–1	LN status, tumour size, BCL2, Her-2, PR, ER, Ki67, p53 and CAIX expression	MV analysis 1.6 (0.8–3.2)	0.2	Reported in text

Table detailing papers which investigated the prognostic role of CAIX on DFS

*IHC* Immunohistochemistry, *BC* Breast cancer, *CT* Chemotherapy, *LN* Lymph node, *LVI* Lymphovascular invasion, *HR* Hormone receptor, *ER* Oestrogen receptor, *PR* Progesterone receptor, *Her-2* Human epidermal growth factor receptor-2, *EGFR* Epidermal growth factor receptor, *AR* Androgen receptor, *CAIX* Carbonic anhydrase IX, *UV* Univariate analysis, *MV* Multivariate analysis, *HR* Hazard ratio, *95%* *CI* 95% confidence interval, *NA* Not available

The pooled HR for univariate analysis was [HR = 1.48, 95% CI (1.19–1.85), *p* = 0.0005] with significant heterogenicity (I^2^ = 61%, *p* = 0.04). The HR for multivariable analysis was [HR = 2.14, 95% CI (1.53–3.01), *p* < 0.0001], with no heterogenicity detected (I^2^ = 0%, *p* = 0.88) (Table 4).

Stratified analysis by study region suggested a poor DFS for three studies with Asian subjects [HR = 2.50, 95% CI (1.57–3.98), *p* = 0.0001] and for five studies from Europe [HR = 1.50, 95% CI (1.15–1.96), *p* = 0.003]. Heterogenicity was observed only among subgroup of Europe (I^2^ = 57%, *p* = 0.05) (Table 4).

There were variations in the antibodies used for IHC in the studies. Five studies (*n* = 778) used M75 antibody and four studies (*n* = 922) used ab50186. Other studies used anti-CAIX antibodies obtained from different suppliers and were used in few studies (*n* = 2), therefore meta-analysis was not carried out. In subgroup analysis by antibody, significant effect of CAIX on DFS was observed in M75 subgroup [HR = 1.51, 95% CI (1.25–1.83), *p* < 0.0001], with no heterogeneity was observed (I^2^ = 0%, *p* = 0.48). A similar association was found in ab50186 [HR = 1.53, 95% CI (1.12–2.10), *p* = 0.008] with moderate heterogeneity (I^2^ = 61%, *p* = 0.05) (Table 4).

Diverse cellular localization was observed between studies. A membranous expression of CAIX was described in five studies (*n* = 734) whereas cytoplasmic staining was only reported in one study. Combination of the membranous and cytoplasmic staining was also reported in two studies (*n* = 479) whereas the rest did not state the staining localization. In subgroup analysis, membranous staining had a significant effect on DFS [HR = 1.69, 95% CI (1.22–2.34), *p* = 0.002]. A significant heterogeneity was detected (I^2^ = 66%,* p* = 0.02) (Table 4).

Eleven studies examined the relationship of various scoring methods and DFS. Percentage of positive cells method was used by three studies (*n* = 584), and intensity of staining was showed in three studies (*n* = 437). While in the remaining four studies (*n* = 861), the scores were calculated as the product of combination of percentage of positive cells and staining intensity. Subgroup analysis of the different scoring revealed a similar significant association between tumoural CAIX expression and DFS in subgroup analysis of percentage of staining cells [HR of 2.57, 95% CI (1.75 − 3.79), *p* < 0.00001], intensity of staining [HR = 1.41, 95% CI (1.13 − 1.76), *p* = 0.002], and the combination of two methods [HR = 1.40, 95% CI (1.13 − 1.74), *p* = 0.002]. Mild heterogeneity was only observed in subgroup analysis of combination of percentage and staining intensity (I^2^ = 37%, *p* = 0.19) (Table 4).

### Analysis of CAIX expression and OS

A total of 16 from the selected 23 studies examined the association between CAIX expression and OS. Three studies with small number of patients were excluded from the analysis. Three studies could not be included in this analysis due to incomplete reporting (Table 3). HR was calculated from available numerical data extrapolated from Kaplan–Meier survival curve and summary table for 3 studies. Based on 10 studies (*n* = 2813), high CAIX expression was statistically significantly associated with a poorer OS [HR = 1.41, 95% CI (1.18 − 1.70), *p* = 0.0002] (Fig. 4). Moderate heterogeneity was detected across these studies (I^2^ = 55%, *p* = 0.02), therefore, Further subgroup analysis was performed.

**Table 3:** Studies characteristics and the impact of CAIX on overall survival

Author(s)	Country	Patients (n)	Median follow up (months)	Cancer death n (%)	Subtype	Antibody for IHC/dilution	Scoring method	High CAIX Expression n (%)	Score range and location	Definition of positive	Tumour stage	Multivariate variables	Hazard ratio (95% CI)	*p-*value	HR estimation
**Alves et al** **2019 **[26]	Brazil	176	73.9	NA	Mixed BC	ab15086 1:200	Percentage and intensity	13 (7.4)	Score 0–6 Membranous	Score ≥ 3	T1-4 N0-3 M0	-	UV analysis NA	0.143	Reported in text
**Ozretic et al** **2018 **[25]	Croatia	64	55.5	10 (15.6)	TNBC	ab15086 1:100	Percentage and intensity	49 (77)	NR Membranous	NA	NA	-	UV analysis NA	0.493	Reported in text
**Noh, Kim, and** **Koo 2014 **[44]	South Korea	334	NA	31 (9.3)	AR + /ER − BC	NA 1:100	Percentage and intensity	96 (28.7)	Score 0–6 Location not stated	Score ≥ 2	T1-3	T stage, LN status, histologic grade and CAIX expression	MV analysis 15.89 (1.82–131.6)	0.010	Reported in text
**Choi, Jung, and** **Koo 2013 **[46]	South Korea	276	67	23 (8.3)	Mixed BC	NA 1:100	Intensity	90 (32.6)	Score 0–3 Cytoplasmic	Score ≥ 2	T1-3 N0-3	-	UV analysis NA	0.195	Reported in text
**Currie et al** **2013 **[52]	New Zealand	87	NA	NA	Mixed BC	NA 1:1000	Percentage and intensity	43 (49)	Score 0–8 Membranous	Score ≥ 3	NA	-	UV analysis NA	0.91	Reported in text
**Betof et al. 2012** [53]	USA	209	99.6	32 (16.6)	Mixed BC based on CT	M75 NR	Percentage and intensity	182 (88)	Score 0–300 Membranous and cytoplasmic	Score ≥ 50	NA	NA	UV analysis 3.77	0.010	Reported in text
**Kaya et al** **2012 **[54]	Turkey	111	110	NA	Group1:HR + , Her-2-ve Group 2:HR-, Her-2 + ve	H-120 1:100	Absent or present	62 (65)	0–1 Membranous	Score ≥ 1	T1-4 N0-3 M0	-	UV analysis NA	0.109	Survival curve
**Beketic-Oreskovic** **et al. 2011 **[23]	Croatia	40	55.8	24 (60)	Mixed BC	NA 1:100	Percentage and intensity	24 (60)	Score 1–3 Membranous and cytoplasmic	Score > 52.5	NA	Necrosis, tumour size, LN status, histological grade and CAIX expression	MV analysis 4.14 (1.28–13.35)	0.018	Reported in text
**Jubb et al** **2010 **[56]	United Kingdom	151	120	NA	Mixed BC	M75 NA	Percentage	49 (32)	0–100 Membranous and cytoplasmic	> 10%	NA	-	UV analysis 0.88 (0.43–1.81)	0.73	Reported in text
**Lancashire et al** **2010 **[48]	United Kingdom	244	67	NA	Mixed BC (*n* = 160)	Abcam 15,086 1:2,500	Absent or present	29 (18.1)	0–1 Membranous	Score ≥ 1	NA	-	UV analysis NA	0.085	Reported in text
**Tan et al** **2009 **[15]	UK and Australia	182	131.9	99 (21.7)	Mixed BC treated with CT (182)	NA	Percentage	59 (14)	0–100 Membranous	≥ 10%	NA	LN status, tumour grade, tumour size and CAIX expression	MV analysis 3.20 (1.79–5.70)	< 0.001	Reported in text
**Kyndi et al** **2008 **[24]	Denmark	945	204	NA	Mixed BC	M75 1:2,500	Percentage	151 (16)	0–100 Membranous	≥ 10%	NA	LN status, tumour size, grade, HR and Her-2 status, menopausal status/systemic treatment	UV analysis 1.30 (1.06–1.60)	NA	Reported In text
**Hussain et al** **2007 **[22]	United Kingdom	144	48	28 (19.4)	Mixed BC	M75 1:100	Intensity and pattern	37 (26)	Score 1–5 Membranous	Score ≥ 2	NR	Vascular invasion and CAIX expression	MV analysis 2.43 (1.07–5.53)	0.035	Reported in text
**Brennan et al** **2006 **[50]	Sweden	400	166.8	NA	Mixed BC 1–3 + ve LN Premenopausal	M75 1:2000	Absent or present	42 (11)	0–1 Membranous	≥ 1%	II	NA	UV analysis NA	0.022	Reported in text
**Generali et al** **2006b **[10]	Italy and UK	169	NR	21 (11.5)	Mixed BC	M75 1:50	Intensity	41 (24)	Score 0–2 Location not stated	Score ≥ 1	T2–4 N0–1	NA	UV analysis NA	0.001	Reported in text
**Chia et al. 2001** [13]	United Kingdom	103	74.4	32 (31)	Mixed BC	M75 1:50	Percentage and intensity	49 (48)	Score 0–300 Membranous	Score ≥ 50	NA	LN status, grade, size, ER, necrosis and CAIX expression	MV analysis 2.61 (1.01–6.75)	0.05	Reported In text

Table detailing papers which investigated the prognostic role of CAIX on OS

*IHC* Immunohistochemistry, *BC* Breast cancer, *CT* Chemotherapy, *LN* Lymph node, *LVI* Lymphovascular invasion, *HR* Hormone receptor, *ER* Oestrogen receptor, *PR* Progesterone receptor, *Her-2* Human epidermal growth factor receptor-2, *EGFR* Epidermal growth factor receptor, *AR* Androgen receptor, *CAIX* Carbonic anhydrase IX, *UV* Univariate analysis, *MV* Multivariate analysis, *HR* Hazard ratio, *95%* *CI* 95% confidence interval, *NA* Not available

As shown in Table 4, the pooled HR for univariate analysis was [HR = 1.27, 95% CI (1.16–1.40), *p* < 0.00001] and heterogeneity was non significant (I^2^ = 10%,* p* = 0.35). The HR for multivariate analysis was [HR = 3.03, 95% CI (1.93–4.77), *p* < 0.00001] and heterogeneity was not reported.

Immunohistochemical staining of CAIX was predominantly performed using the M75 antibody targeting CAIX (*n* = 7 including 2121 patients). The negative association between high CAIX expression in breast cancer and worse OS revealed to be associated with M75 antibody [HR = 1.34, 95% CI (1.14 − 1.57), *p* = 0.0004], with moderate heterogeneity (I^2^ = 40%, *p* = 0.13) (Table 4).

In addition, subgroup analysis based on cellular location was performed. A membranous expression of CAIX was described in five studies (*n* = 1774). Although combination of the membranous and cytoplasmic staining was also reported in two studies (*n* = 360), cytoplasmic staining was only reported in one study (*n* = 276). Whereas two studies did not state the staining localization. Interestingly, the results of the subgroup analysis demonstrate a significant prognostic value of CAIX in membranous location [HR = 1.62, 95% CI (1.21 − 2.17), *p* = 0.001], with significant moderate heterogeneity (I^2^ = 60%, *p* = 0.04) (Table 4).

There was variation in the scoring methods. The most common method being used depending on percentage of antibody-expressing tumour cells (*n* = 3, containing 1278) and combined staining intensity and percentage of positive cells (*n* = 4, containing 822 patients). On the other hand, the least common scoring method was based on staining intensity (*n* = 2). On meta-analysis, statistically significant effect of CAIX on OS was observed when stratified by combination percentage and intensity (HR = 2.70, 95% CI (1.18 − 6.20), *p* = 0.02], with significant heterogeneity (I^2^ = 69%, *p* = 0.02) whereas no association was detected in other subgroups of percentage (HR = 1.51, 95% CI (0.83 − 2.74), *p* = 0.17], with significant heterogeneity (I^2^ = 73%, *p* = 0.02) (Table 4).

**Table 4:** Results of meta-analysis and subgroups of analysis methods, study region, different antibodies, cellular location, and scoring methods reported

Stratified analysis	Number of studies	Number of patients	Pooled HR (95% CI)	*p*-value	Heterogeneity	
					I^2^ (%)	*p-*value
***Recurrence free survival (RFS)***	5	4,578	1.42 (1.32–1.51)	< 0.00001	4%	0.38
***Disease-free survival (DFS)***	10	1,882	1.64 (1.34–2.00)	< 0.00001	49%	0.04
**Analysis methods**						
Univariate	5	875	1.48 (1.19–1.85)	0.0005	61%	0.04
Multivariate	5	1,007	2.14 (1.53–3.01)	< 0.0001	0%	0.88
**Study region**						
Asia	3	706	2.50 (1.57–3.98)	0.0001	0%	0.96
Europe	5	771	1.50 (1.15–1.96)	0.003	57%	0.05
**Antibody for IHC**						
M75 antibody	5	778	1.51 (1.25–1.83)	< 0.0001	0%	0.48
Ab15086 antibody	4	922	1.53 (1.12–2.10)	0.008	61%	0.05
**Cellular location**						
Membranous	5	734	1.69 (1.22–2.34)	0.002	66%	0.02
**Scoring methods**						
Percentage	3	584	2.57 (1.75–3.79)	< 0.00001	0%	0.64
Intensity	3	437	1.41 (1.13–1.76)	0.002	0%	0.39
percentage and intensity	4	861	1.40 (1.13–1.74)	0.002	37%	0.19
***Overall survival (OS)***	10	2,813	1.48 (1.22–1.80)	< 0.0001	59%	0.009
**Analysis methods**						
Univariate	6	2,050	1.27 (1.16–1.40)	< 0.00001	10%	0.35
Multivariate	4	763	3.03 (1.93–4.77)	< 0.00001	0%	0.43
**Antibody for IHC**						
M75 antibody	7	2,121	1.34 (1.14–1.57)	0.0004	40%	0.13
**Cellular location**						
Membranous	5	1,774	1.62 (1.21–2.17)	0.001	60%	0.04
**Scoring methods**						
Percentage	3	1,278	1.51 (0.83–2.74)	0.17	73%	0.02
Percentage and intensity	4	822	2.70 (1.18–6.20)	0.02	69%	0.02

## Discussion

The present systematic review and meta-analysis is the first to examine the prognostic value of CAIX expression in breast cancer. Overall, the results clearly show that high CAIX expression is an adverse prognostic marker in breast cancer independent of the antibody used, tumour localisation, scoring methods and clinical end-points evaluated. Therefore, CAIX expression confirms the hypothesis that hypoxia is an important determinant of clinical outcome in patients with breast cancer.

**Fig. 5:**
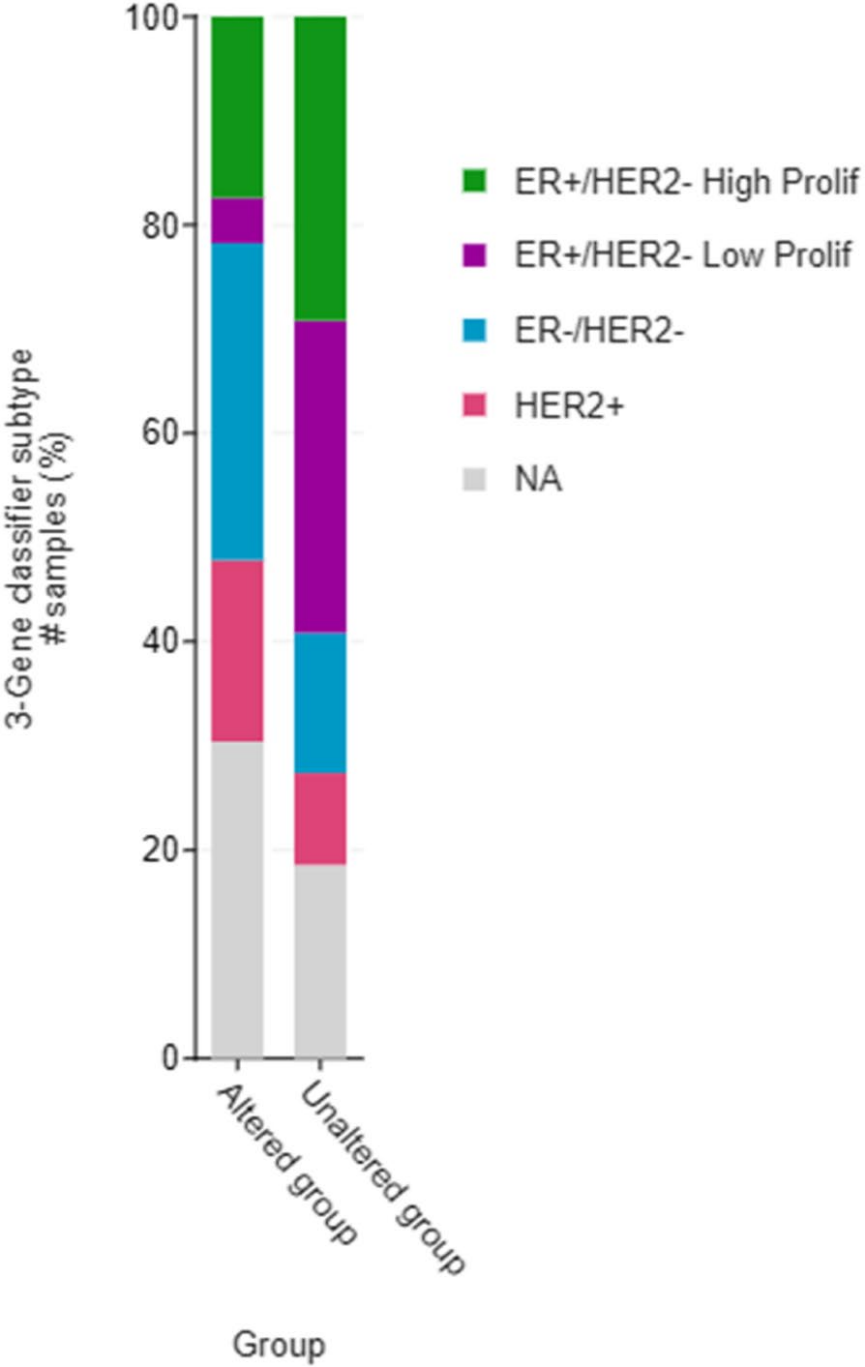
METABRIC breast cancer cohort (*n* = 2051)

Breast cancer is a complex and heterogeneous disease, comprising different histologic and molecular types with different biological features and clinical behaviours. Therefore, we compared the mutation status of CAIX across breast cancer subtypes in the METABRIC breast cancer cohort (*n* = 2051) using online publicly available resource cBioPortal. The CAIX gene was only mutated in 1.1% of cases, however there was a significant association between presence of mutation and breast cancer subtype (*p* = 0.003) as represented in a bar chart (Fig. 5).

The basis of the association between CAIX expression and poor clinical outcome is not clear. However, given that the CAIX enzyme is important in neutralising tumour cell acidification and contributing to extracellular acidification [30]. CAIX is involved in promoting tumorigenesis and leads to a more aggressive phenotype of cancer cells [31]. This can partially be explained by the association between CAIX expression and the induction of metastatic or invasive phenotype by reducing cell adhesion [32], increasing cell invasiveness [33], mobility and migration, stimulating angiogenesis, and activating proteases [34] which could be caused by the reduction in extracellular pH [35]. CAIX also contributes to several specific biological process critical for tumour progression including cell survival, maintenance of cancer stem cell function and chemo and radiotherapy resistance [36]. In addition to serving as a prognostic marker, CAIX may also potentially serve as a promising marker for targeted therapy. In particular, CAIX appears to be highly expressed in breast cancer and has relatively low expression in normal tissues [37–40] and expression is located on the extracellular surface of cell membranes, allowing for efficient targeting by monoclonal antibodies or small molecule inhibitors. Therefore, CAIX constitutes an attractive and promising candidate marker for systemic anticancer therapy. Indeed, carbonic anhydrase inhibitors such as indisulam, a sulfonamide which was investigated in phase II clinical trials, is considered one of the most potent anticancer sulfonamides and has showed high anti-tumour activity in various preclinical tumour models [41]. The combination of CAIX inhibitors with conventional chemotherapy may yield improved efficacy [42]. Also, one of several potent bis-sulfonamide CAIX inhibitors identified by screening 1 million compounds in a DNA- encoded chemical library has exhibited high and specific accumulation in cancer models [43].

It is likely that increased tumour CAIX will promote changes in the metabolic function of stromal and inflammatory cells in close contact with tumour cells such that tumour cells may survive and disseminate [11, 13, 26, 44–46]. However, it is not clear whether increased CAIX expression promotes a specific stromal or inflammatory phenotype or both and further work is required to examine these potential mechanisms of tumour progression.

Similar to HIF‐1α expression, CAIX has been proposed as a marker of an aggressive malignant phenotype in a variety of common solid tumours. However, given that CAIX is less suspectable to degradation, it is perhaps not surprising that there would appear to be a more consistent association with poor clinical outcome compared with HIF-1α [47]. In the present meta-analysis of approximately 8390 patients, CAIX expression was significantly associated and all endpoints: RFS [HR = 1.42, 95% CI (1.32 − 1.51), *p* < 0.00001], DFS [HR = 1.64, 95% CI (1.34–2.00), *p* < 0.00001], and OS [HR = 1.48, 95% CI (1.22 − 1.80), *p* < 0.0001] whereas HIF-1α expression in approximately the same number of patients was only strongly associated with DFS and OS [47]. Moreover, the degree of heterogeneity associated with the HIF-1α expression meta-analysis was greater than that observed for the present CAIX expression meta-analysis. Therefore, the present study would suggest that CAIX expression is more consistently associated with clinical outcomes and may be considered the preferred prognostic marker for tumour hypoxia.

However, in the present study, there was significant heterogeneity in the DFS and OS according to survival analysis, subcellular localization and scoring methods. Therefore, it would appear that careful consideration of technical factors is required when examining the prognostic value of CAIX of patients with breast cancer. Moreover, comparative studies of HIF-1α and CAIX protein expression in the same large mature breast cancer cohort, using optimal methodological approaches, are required to be carried out to confirm this or if whether a combination of these markers should be employed.

With regards to antibody used, two main types of antibodies for IHC were used, M75 and ab50186. The M75 antibody had more consistent prognostic value for DFS and OS. Although different antibody concentrations were reported, subgroup analysis could not be made due to limited number of studies.

The prognostic value of CAIX expression has been reported in both cytoplasmic and membranous locations, however, it is not clear which location has the greater prognostic value. In addition, the relationship between the expression of CAIX in both locations is not clear.

With reference to the scoring methods used, percentage of positive cells, intensity of staining, and combination of percentage of positive cells and staining intensity were consistently associated with DFS whereas only combined percentage and intensity was consistently associated with OS. Therefore, the above potential sources of heterogeneity require further investigation.

### Limitation

There are several limitations of this study. The majority of studies included had relatively small sample sizes which would limit the detection of an association with clinical end-points. Furthermore, the antibodies used, cellular localisation, scoring methods varied considerably in the analysis. Therefore, although we are able to conclude that high CAIX expression is an adverse prognostic factor and that particular antibodies have consistent prognostic value using standard scoring methods in patients with breast cancer, it is not clear what is the optimal prognostic cellular localisation. Further work using, the validated antibodies and scoring methods derived from the present review is required to tease out the importance of CAIX localisation expression. Furthermore, meta-analysis may overestimate associations due to publication bias.

## Conclusion

The present systematic review and meta-analysis clearly shows that high CAIX expression is an adverse prognostic marker in breast cancer independent of the antibody used, tumour localisation and clinical end-point evaluated. Therefore, CAIX expression is consistent with the hypothesis that hypoxia is an important determinant of clinical outcome in patients with breast cancer. Moreover, further work is required to understand the prognostic role of CAIX in the different breast cancer subtypes and stages.

## Data Availability

This review does not contain any studies with human participants or animals performed by any of the authors. All analyses are based on previously published papers.
